# Interferon-related secretome from direct interaction between immune cells and tumor cells is required for upregulation of PD-L1 in tumor cells

**DOI:** 10.1007/s13238-016-0281-6

**Published:** 2016-06-13

**Authors:** Yuan-Qin Yang, Wen-Jie Dong, Xiao-Fei Yin, Yan-Ni Xu, Yu Yang, Jiao-Jiao Wang, Su-Jing Yuan, Jing Xiao, Jonathan Howard DeLong, Liang Chu, Hai-Neng Xu, Xiu-Mei Zhou, Ru-Wei Wang, Ling Fang, Xin-Yuan Liu, Kang-Jian Zhang

**Affiliations:** Xinyuan Institute of Medicine and Biotechnology, Zhejiang Sci-Tech University, Hangzhou, 310018 China; State Key Laboratory of Cell Biology, Shanghai Institute of Biochemistry and Cell Biology, Shanghai Institutes for Biological Sciences, Chinese Academy of Sciences, Shanghai, 200031 China; Sichuan Huiyang Life Science and Technology Corp., Chengdu, 610021 China; College of Life Sciences, Northwest Agriculture and Forestry University, Yangling, 712100 China; Central China Normal University, Wuhan, 430079 China; Department of Pathobiology, School of Veterinary Medicine, University of Pennsylvania, Philadelphia, PA 19104 USA; Department of Radiation Oncology, University of Pennsylvania Perelman School of Medicine, 3400 Civic Center Blvd., Philadelphia, PA 19104 USA; Zhejiang Conba Pharmaceutical Co., Ltd, Hangzhou, 310018 China

**Dear Editor,**

PD-L1, also known as CD274, plays a vital role in tumor cell related immune escape. It can be expressed on the cell surface of many solid tumors (Brahmer et al., 2012) and inhibits T cell proliferation and cytokine production by binding to the T cell surface receptor programmed death 1 (PD-1) or B7-1 (McClanahan et al., 2015). In 2013, targeting PD-1/PD-L1 signaling for cancer immunotherapy was selected as the No.1 scientific breakthrough of the year by the editors of Science. Interferons (IFNs) are a group of pleiotropic cytokines, demonstrated anti-viral, anti-tumor, and immune regulatory functions (York et al., 2015). Type I interferon binds a heterodimeric receptor composed of IFNAR1 and IFNAR2. This activates a canonical JAK/STAT signaling pathway that ultimately induces a set of interferon-stimulated genes to exert its biological activity (Ejlerskov et al., 2015). Recently, PD-L1 was reported to be downstream of IFN signaling in human oral squamous carcinoma, melanoma, and human acute myeloid leukemia blast cells (Chen et al., 2012; Furuta et al., 2014; Kronig et al., 2014).

The tumor microenvironment plays an important role in tumor growth and metastasis. Different components of the tumor microenvironment such as T cells, B cells, NK cells, dendritic cells, mast cells, granulocytes, Treg cells, myeloid derived suppressor cells (MDSC), and tumor associated macrophages (TAM) are recruited by different pathways (Joyce and Fearon, 2015). Tumor cells have been shown to upregulate PD-L1 after interacting with infiltrating immune cells (Cho et al., 2011; Hou et al., 2014), but the mechanism by which this occurs is not well understood. In this study, we found that PD-L1 upregulation in tumors was dependent on direct interaction with immune cells and was driven by a secreted factor such as type I interferon after cell-cell contact.

Previous studies have demonstrated a positive correlation between tumor-infiltrating immune cells and elevated PD-L1 expression in tumor cells, but the mechanism by which this occurs is poorly understood. To investigate this, we co-cultured murine B16F10 melanoma cells with syngeneic splenocytes for 48 h. In addition, to determine whether direct cell contact is required for immune cell-mediated PD-L1 expression, the two types of cells were separated by a transwell-membrane that blocked their direct cell-cell interactions. Furthermore, another condition was tested in which B16F10 cells and immune cells were co-cultured in the plate and B16F10 cells were cultured in the transwell insert (Fig. 1A). Then the non-adherent immune cells were removed and B16F10 cells were harvested and analyzed for PD-L1 expression by flow cytometry. PD-L1 was more highly expressed in B16F10 cells that were co-cultured with splenocytes than in those cultured alone (Fig. 1B). However, PD-L1 expression was not increased in B16F10 cells separated from the splenocytes by a transwell membrane. We also found that a B16F10-splenocyte co-culture was able to induce PD-L1 in tumor cells separated from the co-culture by a transwell membrane (Fig. 1B). These effects were also observed in PD-L1 mRNA level changes by qPCR (Fig. 1C). These results suggested that active factors were secreted into the supernatant after the direct cell-cell interaction that was able to induce PD-L1 expression in tumor cells.

**Figure 1:**
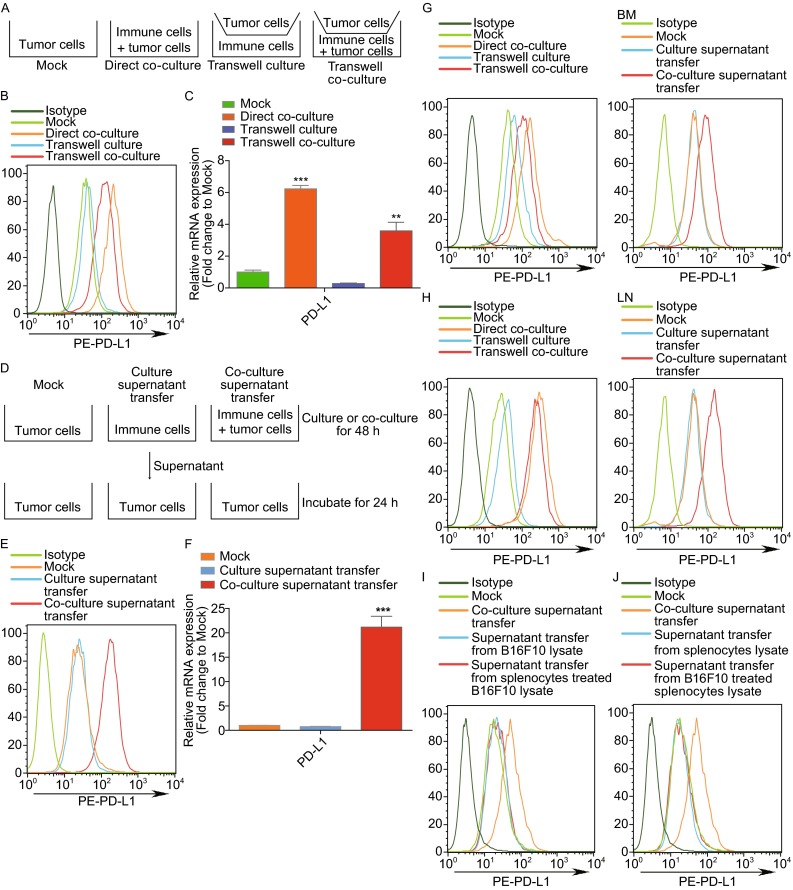
**Upregulation of PD-L1 in tumor cells required secreted factors from living cells after direct cell-cell interactions**. (A) Schematic diagram of the different co-culture conditions of tumor cells and immune cells (primary splenocytes, bone marrow (BM)-derived cells, or lymph node (LN)-derived cells). Tumor cells were directly mixed with immune cells (Direct co-culture) or not (Mock). In the transwell co-culture system, tumor cells were seeded onto the upper insert with the lower compartment containing immune cells (Transwell culture) or a mixture of immune cells and tumor cells (Transwell co-culture). (B and C) Expression of PD-L1 in B16F10 cells was determined by flow cytometry (B) and RT-qPCR (C). (D) Schematic diagram for treatment of tumor cells with supernatant from co-cultured tumor cells and splenocytes (Co-culture supernatant transfer), tumor cells alone (Mock) or splenocytes alone (Culture supernatant transfer) as control groups. (E and F) Expression of PD-L1 was determined by flow cytometry (E) and RT-qPCR (F). (G and H) PD-L1 expression was determined by flow cytometry in B16F10 cells by co-culturing with BM (G) or LN cells (H). (I) B16F10 tumor cells were treated for 24 h with supernatant from a 48 h culture of live B16F10 cells (Mock), live splenocytes with B16F10 lysate (supernatant transfer from splenocytes treated B16F10 lysate), live B16F10 cells and live splenocytes (Co-culture supernatant transfer), or B16F10 cell lysate (supernatant transfer from B16F10 lysate). (J) Similarly, B16F10 tumor cells were treated with supernatant from live B16F10 cells with splenocyte lysate (supernatant transfer from B16F10 treated splenocytes lysate), or splenocyte lysate (supernatant transfer from splenocytes lysate). PD-L1 expression was measured by flow cytometry

To identify whether the regulation of PD-L1 was indeed driven by a secreted factor, B16F10 cells and splenocytes were co-cultured for 48 h. The supernatant was collected and centrifuged, and then used to treat B16F10 cells independently. The corresponding supernatant derived from B16F10 cells and splenocytes alone was also used to treat B16F10 cells as control groups (Fig. 1D). After 24 h, B16F10 cells treated with supernatant from the co-culture expressed more PD-L1 than cells treated with supernatant from the control mono-cultures (Fig. 1E and 1F). In addition, co-cultures of B16F10 cells with bone marrow (BM)-derived cells (Fig. 1G) or lymph node (LN)-derived cells also upregulated PD-L1 expression (Fig. 1H). To determine whether a similar effect would be seen in other types of cancer cells, additional studies on MC38 and Hepa1-6 cells were performed and the same result was obtained (Fig. S1).

Some evidence suggests that cellular components such as tumor cell-derived antigen or other cellular components may also induce PD-L1 expression. To examine these possibilities, we tested whether B16F10 cell-related tumor antigen can stimulate immune cells to secrete type I IFN and whether immune cell-derived components can stimulate tumor cells to upregulate PD-L1. Thus, living immune cells were cultured with B16F10 lysate and live B16F10 tumor cells were cultured with splenocyte lysate. We found that neither lysate can induce PD-L1 expression (Fig. 1I and 1J). These results demonstrated that cell lysate is not sufficient to upregulate PD-L1, suggesting that living cells are required.

It has been reported that PD-L1 expression is induced by IFN signaling. Here we confirmed that the interferon signal was involved. The mRNA expression level of interferon stimulated genes such as IRF7 and ISG15 was significantly upregulated by the supernatant derived from the co-culture of B16F10 cells with bone marrow cells, lymph node cells, or splenocytes (Fig. 2A–C). Moreover, the phosphorylation of STAT1 and STAT3 were increased by supernatant treatment (Fig. 2D). Further, it was observed that co-culture of B16F10 and immune cells contributed to more IFN-α and IFN-β release in their supernatant (Fig. S2A and S2B). It is known that interferons (IFN-α, IFN-β, and IFN-γ) induce PD-L1 in tumor cells and the above data suggested that interferon signaling may mediate PD-L1 expression in this system. To determine whether the expression of PD-L1 can be induced by type I IFN in B16F10 cells, we treated B16F10 cells with IFN-β and the flow cytometry results showed that IFN-β can induce PD-L1 in a concentration-dependent manner (Fig. S3A). Additionally, by using human fibrosarcoma 2fTGH cells (*ifnar2+*/*+*) and the mutant U5A cells (*ifnar2-*/*-*) that lack of type I interferon receptor subunit ifnar2, we found that IFN-α induced PD-L1 in 2fTGH cells, but failed to induce PD-L1 in U5A cells (Fig. S3B). These data suggested that the induction of PD-L1 by co-culture supernatant may be interferon receptor dependent. To test this hypothesis, anti-IFNAR1 antibody was added to the co-culture medium. We found that the neutralizing antibody reduced splenocyte-induced PD-L1 expression in B16F10 cells (Fig. 2E). The suppression of PD-L1 expression by anti-IFNAR1 was further confirmed by transwell and supernatant transfer assays (Fig. 2F and 2G). Similarly, these results were observed in BM and LN cell co-culture experiments (Fig. S4). However, in splenocytes and LN cell co-culture, anti-IFNAR1 antibody only partially blocked PD-L1 expression, suggesting that other secreted factors were involved in stimulating the PD-L1 expression. After testing the different supernatant by cytokines/chemokines microarray, higher IFN-γ, IL-6 (well proved for PD-L1 regulation), and G-CSF, Leptin, MIG, MCP-5, MIP-1a (firstly observed here may related to PD-L1 regulation) expression were found in the supernatant from co-culture of B16F10 and immune cells compared to supernatant from B16F10 (Figs. 2H, S5A, and S5B). Additionally, we found that after adding the anti-IFNAR1 antibody, PD-L1 expression in B16F10 cells were unable to response to type I IFN stimulation (Fig. S3C). However, IFN-γ still induced PD-L1 expression in B16F10 cells treated with and without anti-IFNAR1 antibody (Fig. S3D). The above findings suggested a working model in which direct contaction between immune cells and tumor cells induces the secretion of factors including type I/II interferons, then these factors promote PD-L1 expression in both contacted and uncontacted tumor cells (Fig. 2I).

**Figure 2:**
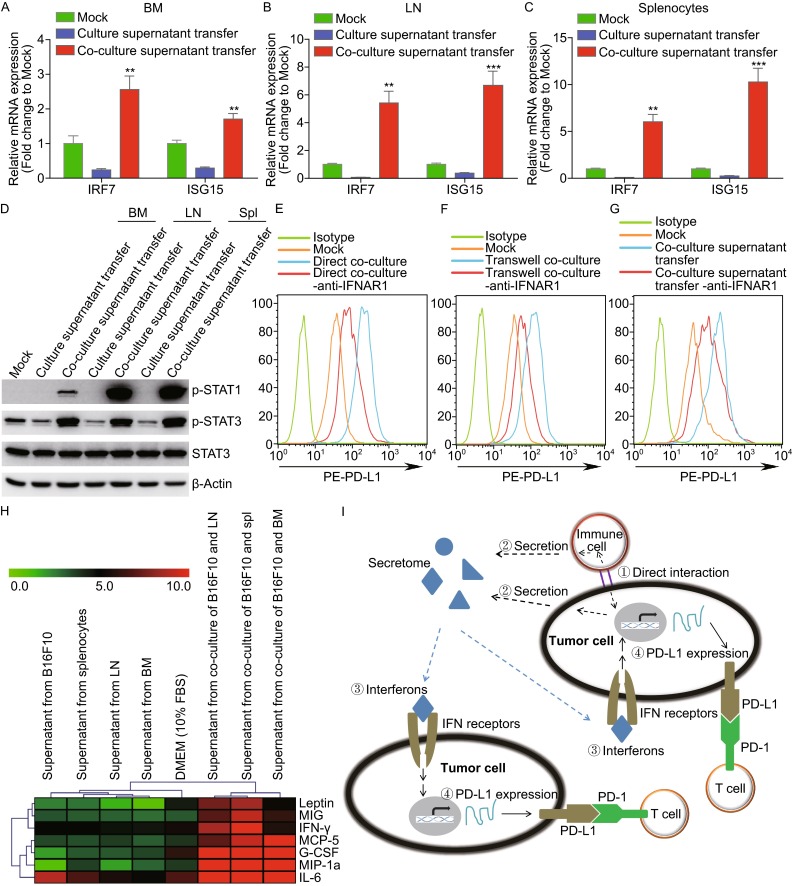
**Upregulation of PD-L1 in tumor cells was dependent on IFNAR1 signaling**. (A–C) IRF7 and ISG15 mRNA level in B16F10 cells were determined, which were treated for 24 h with co-culture supernatant from B16F10 cells and bone marrow (A), or lymph nodes derived cells (B) or splenocytes (C), respectively. (D) After treated with the similar above supernatant in (A–C) for 15 min, the phosphorylation of STAT1 and STAT3 were determined. (E–G) B16F10 cells were co-cultured with splenocytes in three different co-culture systems with or without the 20 µg/mL anti-IFNAR1 antibody. PD-L1 expression was measured by flow cytometry in direct co-culture system (E), transwell co-culture system (F), and supernatant transfer system (G). (H) Heat map demonstrated relative abundance of cytokines in different cell co-culture supernatant. (I) Schematic model of PD-L1 induction in tumor cells by the interaction between immune and tumor cells. Immune cells directly contact tumor cells (➀ Direct interaction). Then the secretome is actively secreted into the supernatant (➁ Secretion). Interferons, one type of factors in the secretome, bind to IFN receptors (➂ Interferons) and further upregulate endogenous PD-L1 expression in both contacted tumor cells and uncontacted tumor cells (➃ PD-L1 expression)

PD-L1 is expressed in multiple different tumor cells that suppress anti-tumor immunity in the tumor microenvironment. By binding to its receptor PD-1 on T cells, PD-L1 inhibits the T cell-mediated immune response. Targeting PD-L1/PD-1 signaling has been shown to be a highly efficacious cancer immunotherapy (Brahmer et al., 2012; Topalian et al., 2012). Several signaling pathways have been shown to upregulate PD-L1 in tumor cells, including NF-κB, AKT, IFN, and IL-6 (Chen et al., 2012; Jin et al., 2013; Gowrishankar et al., 2015; Lastwika et al., 2016). However, the factors promoting elevated PD-L1 expression in the tumor microenvironment have not been thoroughly elucidated.

In this study, we demonstrated that tumor cell PD-L1 expression is elevated by co-culture with immune cells, and this effect is dependent on the direct interaction between tumor and immune cells. A previous study revealed that CD11b^+^ myeloid cells adhered to the surface of tumor cells to induce tumor PD-L1 expression through the p38 pathway (Noh et al., 2015). Here, we found that direct cell-cell interactions are required for the production of the secreted factors that induce PD-L1 expression in B16F10 cells. Additionally, the secretome including IFNs induce the PD-L1 expression. However, PD-L1 expression can be partially blocked with anti-IFNAR1 antibody in splenocytes and LN cells co-culture models. These data suggested the possible contribution of other molecules in the regulation of PD-L1 expression by protein microassay. But in our study, which cell population secreted IFNs and which type of immune cells play a key point for cell-cell contaction still need further study.

On the basis of others' and our studies, we think that CD11b^+^ cells may phagocytose tumor cells to trigger production of type I IFN in STING signaling-dependent manner.

Blockade of PD-1/PD-L1 signaling with monoclonal antibodies has been demonstrated to be a promising new immunotherapy. However, this therapy has a limited role on extending survival time and can lead to inflammatory side effects called immune-related adverse events (irAEs) in other normal tissues (Brahmer et al., 2012). Our findings suggest an alternative way to find specific inhibitors to limit PD-L1 expression in tumor cells by blocking its up-stream induced signaling. Such screened inhibitors could be used in combination with PD-L1 antibodies to achieve better effect for cancer therapy.

## Electronic supplementary material

Below is the link to the electronic supplementary material.

Supplementary material 1 (PDF 803 kb)

Supplementary material 2 (PDF 102 kb)
