# Supplementary material for: Interferon-related secretome from direct interaction between immune cells and tumor cells is required for upregulation of PD-L1 in tumor cells
Source: Protein Cell. 2016 Jun 13;7(7):538–43. doi: 10.1007/s13238-016-0281-6 (PMC4930771; doi:10.1007/s13238-016-0281-6)
Supplement: Supplementary file 1 — Supplementary material 1 (PDF 803 kb) [file 13238_2016_281_MOESM1_ESM.pdf]

Supplementary Figures

Fig. S1

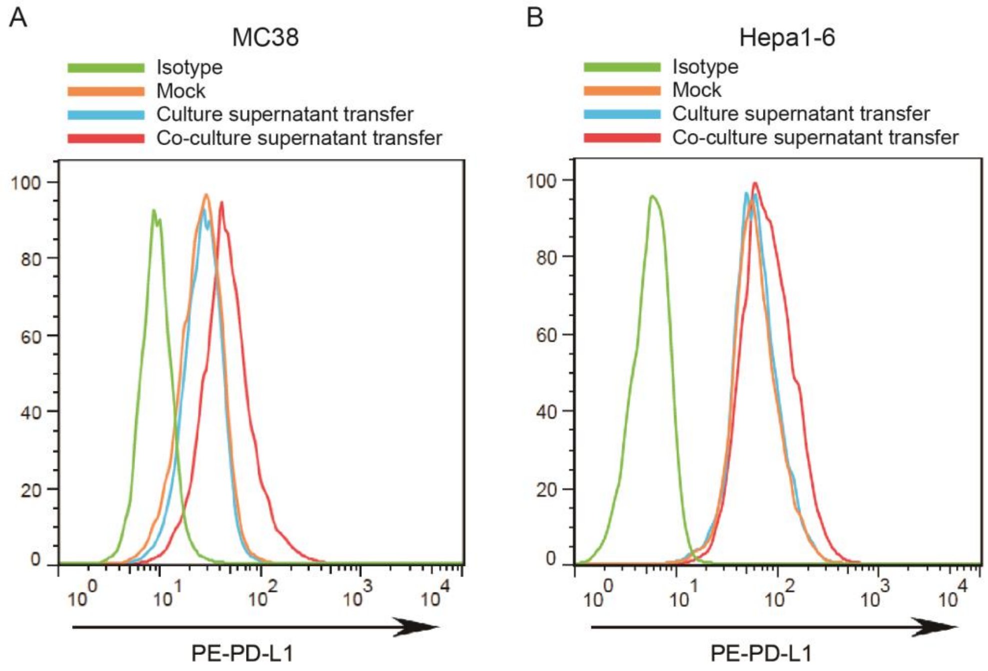

Fig.S1

Supernatant-mediated induction of PD-L1 on other tumor cell lines. MC38 cells or Hepa1-6 cells were cultured with splenocytes and the supernatants from these co-cultures were added to mono-cultures of these tumor cells as in Fig 3A. PD-L1 expression on MC38 cells (A) and Hepa1-6 cells (B) was analyzed by flow cytometry.

Fig. S2

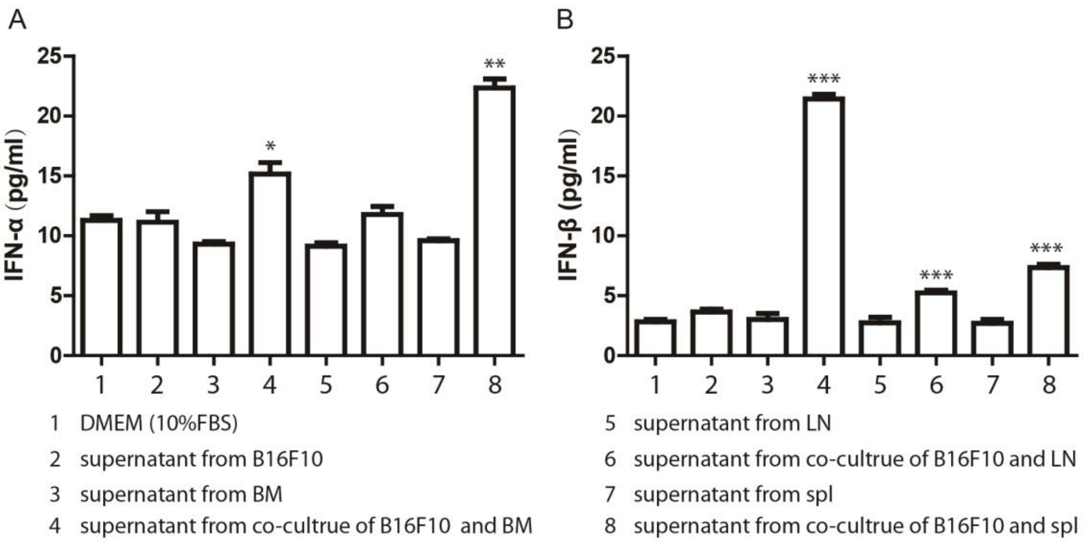

Fig.S2

B16F10, BM, LN and splenocytes culture along, and B16F10 co-culture with BM, LN and splenocytes. Then the supernatant were collected after 48 hr. IFN-α (Fig. S2A) and IFN-β (Fig. S2B) was quantified by ELISA in different supernatants.

Fig. S3

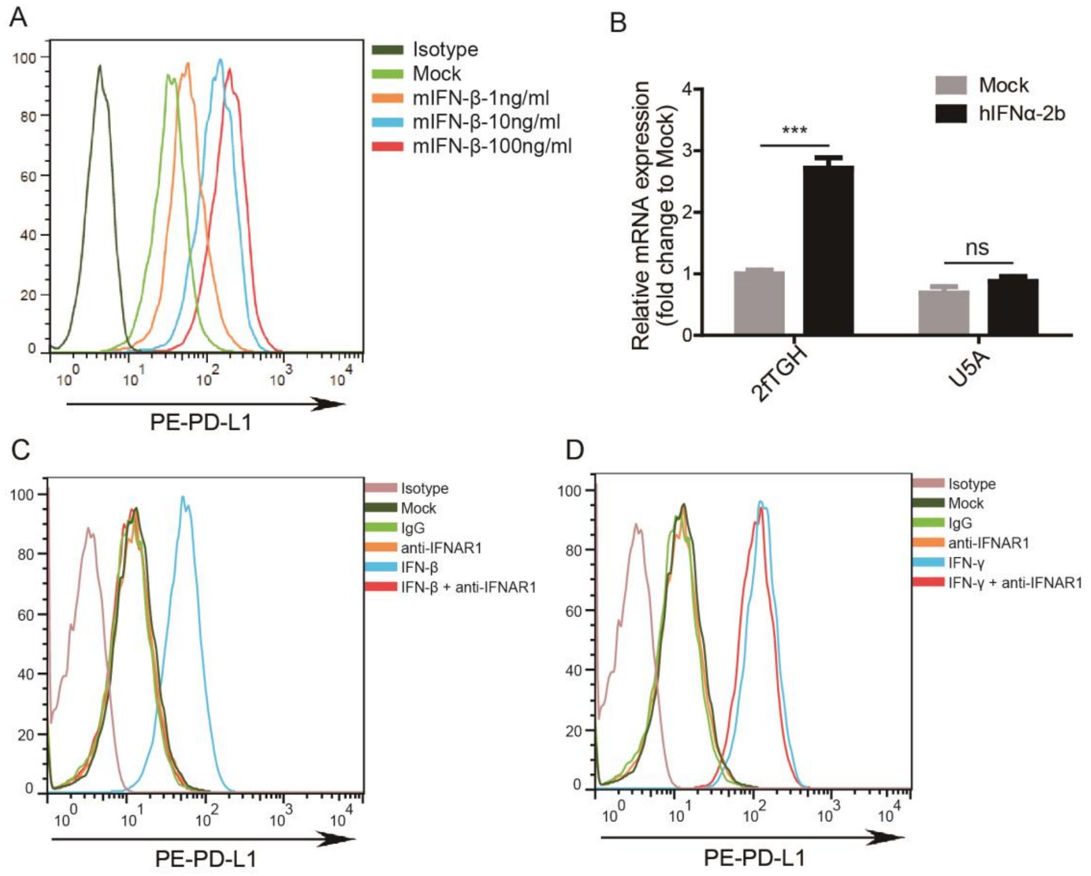

Fig.S3

Type I interferon induced the expression of PD-L1 in multiple tumor cell lines. (A) PD-L1 was induced in a titratable fashion by different concentrations (1 ng/ml, 10 ng/ml, 100 ng/ml) of mouse IFN-β, as assessed by flow cytometry. (B) Human fibrosarcoma 2fTGH cells (*ifnar2+/+*) and U5A(*ifnar2-/-*) cells were stimulated by human IFN-α at 10ng/ml. PD-L1 expression was observed by RT-qPCR . (C and D) Both type I and II interferon induced PD-L1 expression in B16F10 cells. B16F10 cells were treated by mouse IFN-β, mouse IFN-β+IFNAR1, then PD-L1 was detected by flow cytometry. Similarly, PD-L1 expression was observed after cells treated with mouse IFN-γ, mouse IFN-γ+IFNAR1.

Fig. S4

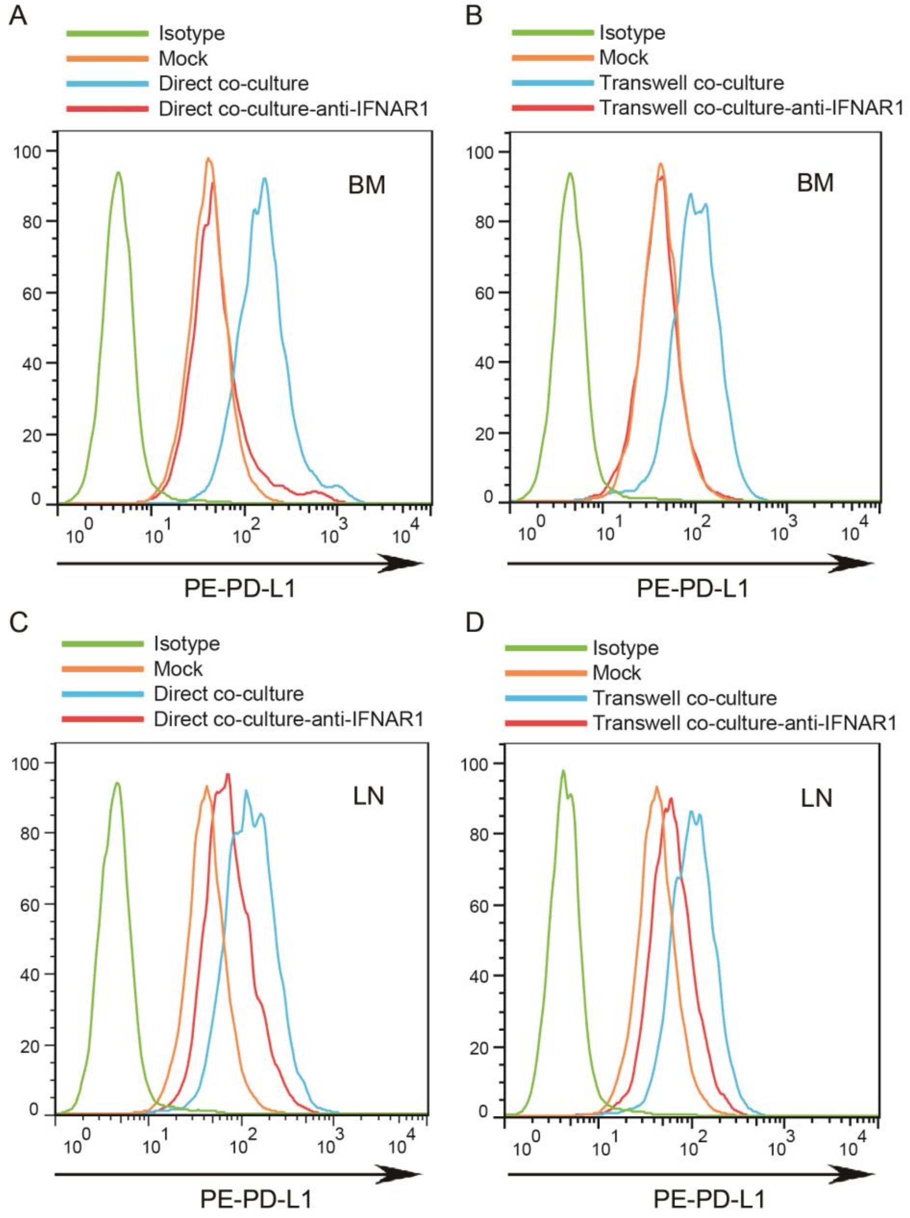

Fig.S4

Upregulation of PD-L1 in tumor cells was dependent on IFNAR1 signaling in BM and LN cell co-culture system. Similarly to Fig. 2E and 2F, but using other sources of immune cells, B16F10 cells were co-cultured with primary BM or LN cells in different co-culture systems with or without the addition of 20ug/mL anti-IFNAR1 antibody. PD-L1 expression was measured by flow cytometry in the direct co-culture system (A and C) or the transwell co-culture system (B and D).

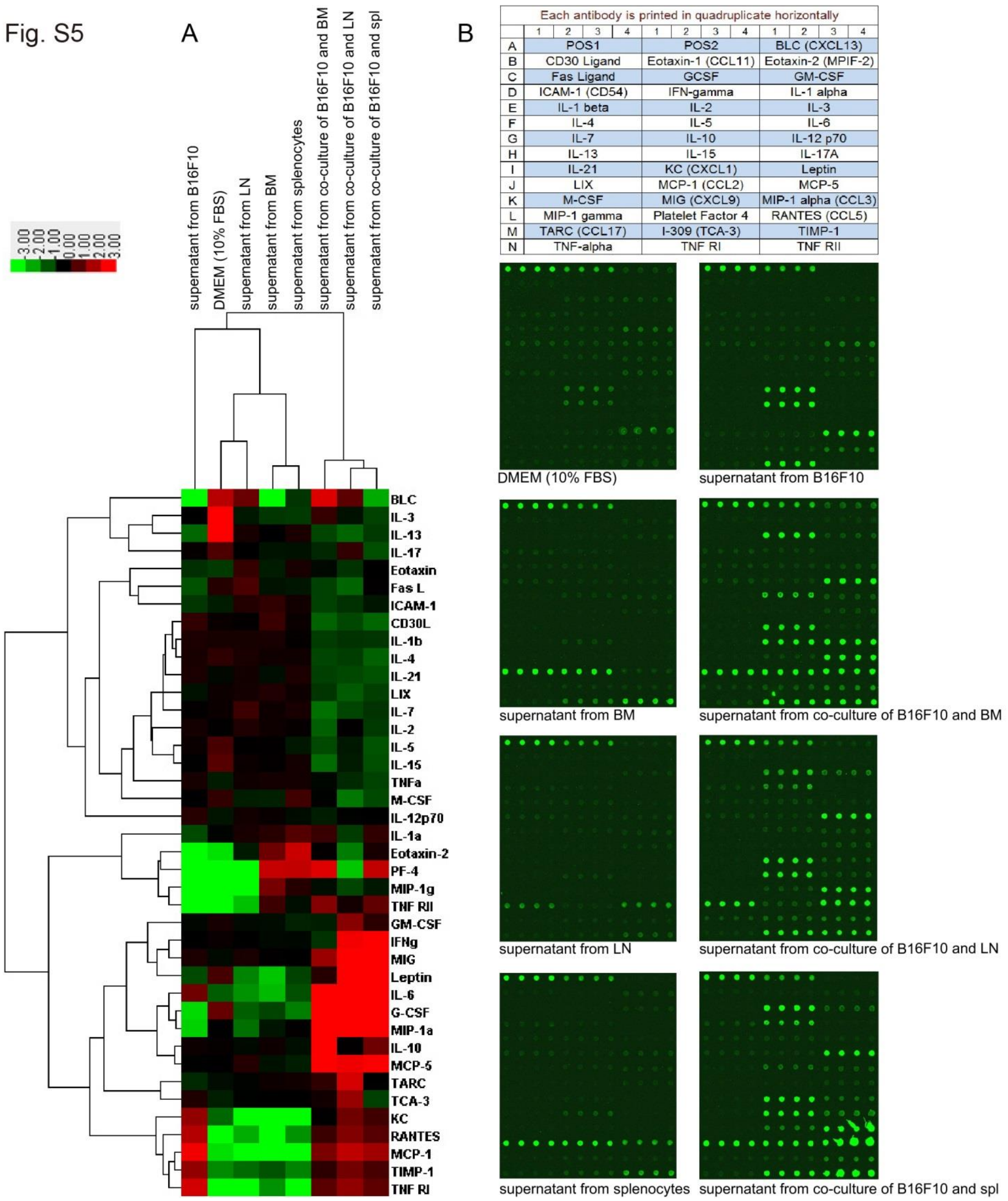

Fig.S5

Detection the secreated factors in different supernatants. (A) Heatmap data for the different sectors from collected supernatants (B16F10, BM, LN and splenocytes single culture as well as B16F10 co-culture with BM, LN and splenocytes for 48 hours). (B) Detailed information on Cytokines/chemokines microarray. The arrangement for the targeting cytoke/chemokines in the chip(upper table) and the scanned fluoresced signaling for the chip (bottom).
