# Supplementary material for: Interferon-related secretome from direct interaction between immune cells and tumor cells is required for upregulation of PD-L1 in tumor cells
Source: Protein Cell. 2016 Jun 13;7(7):538–43. doi: 10.1007/s13238-016-0281-6 (PMC4930771; doi:10.1007/s13238-016-0281-6)
Supplement: Supplementary file 2 — Supplementary material 2 (PDF 102 kb) [file 13238_2016_281_MOESM2_ESM.pdf]

Supplementary Materials

Materials and methods

Cells and Reagents

The mouse melanoma B16F10 cells were a gift from Prof. Hongyan Wang . The mouse coloretical cancer MC38 cells were kindly provided by Prof. Serge Fuchs. The mouse hepatic cancer Hepa1-6 cells were were obtained from Cell Bank of the Type Culture Collection of Chinese Academy of Sciences (Shanghai, China). All cells were cultured in their corresponding completed medium according to ATCC instructions.

In this study, the following antibodies were used: phycoerythrin (PE)- conjugated anti-mouse PD-L1 (clone 10F.9G2, catalog number:124308), PE-conjugated isotype controls and anti-mouse IFNAR1 (clone MAR1-5A3, catalog number:127322) were obtained from Biolegend Inc.; Phospho-STAT1 (Tyr701, catalog number :9167), Phospho-STAT3 (Tyr705, catalog number:9145) and STAT3 (catalog number:4904) were acquired from Cell Signaling Technology. 1 um cell culture insert (catalog number:353102) and six-transwell plate (catalog number:353502) were purchased from BD Biosciences.

Mouse

C57BL/6 strain(7-9 week old) were purchased from Shanghai Laboratory Animal Center (SLAC). All the animal experiments were performed according to the regulations from the U.S. Public Health Service Policy on Humane Care and the Use

of Laboratory Animals.

Isolation of different primary tissue cells

The bones were isolated from the rear legs of mice and washed with Dulbecco's modified eagle medium (DMEM) medium. Bone marrow cells were flushed out via a 1 ml syringe. Mesenteric, brachial, and inguinal lymph nodes were isolated and divided into a single cell suspension using a 100 um cell strainer. Spleens were processed in a similar manner. All primary cells were purified by removing the red cell via red blood cell lysis buffer. The cells were then used for co-culture system with tumor cells.

Cells co-culture

Tumor cells and immune cells were co-cultured in 6-well plates at a density of 3×10^5 and 3×10^6, respectively. After 48 hours, the immune cells were washed off and the attached tumor cells were collected for analysis. In the transwell system, 2×10^5 tumor cells were cultured in the inserted chamber. Similarly, immune cells were cultured alone or co-cultured with tumor cells. For experiments on the supernatant with secreted factors from the co-culture system, tumor cells and immune cells were co-cultured in 6-well plates. After 48 hours, supernatants were collected and centrifuged at 12,000 g for 3 minutes. Then supernatants were added to tumor cell cultures for 24 hours. Then tumor cells were detached and collected for analysis.

Western Blot

Cells were lysed in cell lysis buffer and vortexed at 4 ℃ for 10 minutes, then centrifuged at 12,000 g for 10 minutes. Total proteins were quantified and separated by 10 % SDS-PAGE and transferred to PVDF membrane. After blocking with 5% non-fat milk, membranes were immunoblotted with antibodies over night, followed by horseradish peroxidase (HRP)-linked secondary antibodies.

FACS assay

Cells were harvested and then incubated with anti-PD-L1 antibody in a 1:100 dilution in PBS for 30 minutes in the dark at room temperature. Cells were analyzed on a FACS Calibur flow cytometer (BD, Grand Island, NY) and the results was analyzed with FlowJo 7.6 software (Tree Star, Inc., Ashland, OR).

Real-time quantitative PCR (RT-qPCR)

Total RNA was isolated from cells with Trizol (CWbio, Beijing, China). 1 μg total RNA was used for reverse transcription qPCR using ReverTra Ace qPCR RT Kit (TOYOBO, Code No. FSQ-101, Japan). RT-qPCR was performed by Bio-Rad CFX-96 using SuperReal PreMix Plus SYBR Green (Tiangen biotech company, Cat: FP205, Beijing) according to the manufacturer's instructions. The following primers were used:

PD-L1-forward	TCAGCTACGGTGGTGCGGACT
PD-L1-reverse	AGCTTCTGGATAACCCTCGGCCT

IRF7-forward	AATTCCTACCTGTTACCA
IRF7-reverse	ATGCTACTACTCTGTGAT
ISG15-forward	GGTGTCCGTGACTAACTCCAT
ISG15-reverse	TGGAAAGGGTAAGACCGTCCT
GAPDH-forward	ATGCTACTACTCTGTGAT
GAPDH-reverse	GTAGACTCCACGACATACTCAGC

ELISA assay

Mouse IFN-α and IFN-β levels in the different cell cultured supernatants were measured by ELISA kit (R&D Systems) according to the protocols of the manufacturer.

Cytokines/chemokines microarray

The supernatants were collected after 48 hr cell culture. The samples were quantified by a Quantibody Mouse Inflammation Array I kit (RayBiotech, Inc. Norcross, GA; Cat. No. QAM-INF-1) according to the manufacturer's instructions.

Statistical analysis

Comparison between groups were performed by student’s t-test or one-way analysis of variance using Prism (GraphPad). *$P < 0.05$; **$P < 0.01$; ***$P < 0.001$; n.s., not significant.

Abbreviations

Programmed death-ligand 1, PD-L1

Programmed cell death protein 1, PD-1

Cluster of differentiation 274, CD274

Interferon, IFN

Bone marrow, BM

Lymph node, LN

Interferon regulatory factor 7, IRF7

Interferon stimulated gene 15, ISG15
